# Ultrasound assessment of hepatomegaly and metabolically-associated fatty liver disease among a sample of children: a pilot project

**DOI:** 10.3389/fped.2025.1491342

**Published:** 2025-04-28

**Authors:** Bárbara L. Riestra-Candelaria, Wilma Rodríguez-Mojica, Camille Vélez-Morell, Claudia Ramírez-Marcano, Ariana Alvarado-Castillo, Gabriel Camareno-Soto, Loida A. González-Rodríguez

**Affiliations:** ^1^ Department of Anatomy and Cell Biology, School of Medicine, Universidad Central del Caribe, Bayamón, Puerto Rico; ^2^ Department of Radiological Sciences, School of Medicine, University of Puerto Rico, San Juan, Puerto Rico; ^3^ Medical Student, School of Medicine, Universidad Central del Caribe, Bayamón, Puerto Rico; ^4^ Department of Medicine-Endocrinology, Diabetes and Metabolism Division, School of Medicine, University of Puerto Rico, San Juan, Puerto Rico

**Keywords:** metabolic-associated fatty liver disease, pediatric, liver steatosis, hepatomegaly, obesity, overweight, children

## Abstract

**Introduction:**

Obesity in children is a global health crisis, with 46% of children in Puerto Rico classified as overweight or obese based on Body Mass Index. This condition is linked to serious comorbidities, including early-onset type 2 diabetes, hypertension, and Metabolic-Associated Fatty Liver Disease (MAFLD), the most common liver disease in U.S. children. This study examines the relationship between body weight, liver size, and texture in children from Puerto Rico.

**Methods:**

A craniocaudal right liver lobe (RLL) measurement was performed using a panoramic ultrasound image. RLL length and liver texture were assessed based on fat infiltration. BMI was calculated to classify participants into healthy and unhealthy weight groups, and waist circumference (WC) was compared. Statistical analyses, including Shapiro–Wilk, Student's t-tests, ANOVA, and *post hoc* Tukey HSD, were conducted with significance at *p* ≤ 0.05.

**Results:**

Forty-three children aged 7–19 years were recruited. Significant differences were observed in liver size and texture between healthy and unhealthy weight groups: RLL length (*p* = 0.003), WC (*p* < 0.001), and BMI (*p* < 0.001). Obese children had significantly larger RLL and WC than healthy-weight group (*p* = 0.02; *p* < 0.001). More children in unhealthy weight group exhibited hepatomegaly (*n* = 12) and fat infiltration (*n* = 15).

**Discussion:**

The findings indicate that large liver and MAFLD are common among children with overweight and obesity, suggesting liver changes related to obesity begin early in life. Strategies to maintain a healthy weight in children are essential to reduce the risk of chronic diseases and potential disabilities in adulthood.

## 1 Introduction

Children with overweight and obesity have become a global epidemic with the highest prevalence among the Hispanic population (1, 2). In Puerto Rico, 46% of the children are overweight or obese (unhealthy weight) according to their Body Mass Index (BMI). Children with obesity face a significantly increased risk of developing various long-term comorbidities, including the emergence of metabolic-associated fatty liver disease (MAFLD). MAFLD, previously known as liver steatosis, is the primary global chronic liver disease, primarily affecting children with overweight and obesity (3). MAFLD is a precursor to severe chronic liver conditions such as steatohepatitis and fibrosis (4–7). Furthermore, it is anticipated that within the next decade, MAFLD will become a predominant cause of liver complications among children (6, 8).

Monitoring liver function tests is known to have limitations as they may yield normal results despite MAFLD (9–11). The gold standard for detecting hepatic diseases is a liver biopsy, a procedure known for its invasiveness, pain, cost, and potential risks to patients (12, 13). As a non-invasive alternative tool, ultrasound creates real-time images that offer a valuable means to measure liver parameters and assess anatomical liver changes without known adverse effects, which is particularly important for children patients (14, 15).

MAFLD is a multisystemic disease, and without correct and timely treatment, it will lead to significant and longstanding morbidity and mortality during adulthood. Even though the prevalence of MAFLD is higher in children with overweight and obesity, its presence in children with healthy weight is worrisome (1, 16, 17). Although obesity has been associated with MAFLD, it is not clear how body weight is related to the size and texture of the liver in the children population.

This study aimed to explore the relationship between body weight and children liver health, using sonographic measurements and findings focusing on right liver lobe length (RLL) and liver texture within a sample of children from Puerto Rico. We predicted that (1) ultrasound would be a useful tool to diagnose early changes in the texture and size of the children's livers and (2) most children diagnosed with large, fatty-infiltrated livers would be children with unhealthy weight.

## 2 Methods and materials

### 2.1 Participants

The study population included children between 7 and 19 years of age recruited from the Pediatric Endocrinology Clinic of the University Center of Integral and Complementary Medicine of Puerto Rico (CUMIC). Inclusion criteria included age > 7 and <21 years and child and parent consent to perform the sonogram. Exclusion criteria included (1) children unable to follow instructions for 20–30 min, remain lying on the table for an extended period, and hold their breath for several seconds; (2) Children or parents who do not want to consent to participate in the study; (3) Foster Kids; (4) children previously diagnosed with diabetes mellitus type 2 and (5) pre-existing hepatic and renal disease. The CDC BMI Percentile Calculator for Children and Teens classified participants into healthy and unhealthy weight groups. For this study, the unhealthy weight group was overweight and obese children. Healthy weight was defined as a BMI above the 5th percentile to less than the 85th percentile for children and teens of the same age and gender. Overweight is defined as having a Body Mass Index (BMI) at or above the 85th percentile but below the 95th percentile for children and teens of the same age and gender, while obesity is defined as a BMI at or above the 95th percentile. Waist circumference (centimeters), weight (pounds), and height (inches) were measured for each participant.

This study was approved by the IRB office of the Universidad Central del Caribe, protocol number 2021-13.

### 2.2 Sonography evaluation

A portable GE Logiq E Ultrasound Machine with a curve transducer was used to perform sonographic images of the liver, spleen, and kidneys. Images of the spleen and kidneys were used to compare them with the texture of the liver and determine fat infiltration using the Rumack (15) scales. Panoramic images of the liver were taken to measure it craniocaudally, as recommended by a previously published article by Riestra (10). A certified radiologist evaluated sonographic images. Hepatomegaly was diagnosed by the radiologist using pediatric parameters by age range published in Radiology Assistant (18).

### 2.3 Statistical analysis

Data analysis included a two-tailed paired *T*-test to compare groups, with statistical significance set at *p* < 0.05. Normality was assessed using the Shapiro–Wilk test. A one-way ANOVA was conducted to compare means among multiple groups, followed by Tukey's *post hoc* test for pairwise comparisons when significant differences were detected. The unhealthy weight group was stratified into overweight and obese.

## 3 Results

### 3.1 Participants

Forty-three children from Puerto Rico between the ages of 7 and 19 were recruited to undergo sonographic views. There were no participants over 19 years old. A panoramic craniocaudal ultrasound image of the right liver lobe (RLL) was performed to assess liver length and texture. The mean age of the participants was 13.2 ± 3.5 years, with 56% being female.

### 3.2 BMI

According to children's BMI classification, 22 children were in the healthy weight category, while the other 21 were in the unhealthy group. They were stratified into overweight (10; BMI above the 85th percentile and below the 95th percentile) and obese (11; BMI at or above the 95th percentile). The mean BMI in the healthy group was 19.8 kg/m^2^, while that of the unhealthy group was 28.7 kg/m^2^. Stratification of the unhealthy weight group showed an average BMI of 26.2 kg/m^2^ in the overweight group and 31.0 kg/m^2^ in the obese group.

### 3.3 Waist circumference

**Figure 1:**
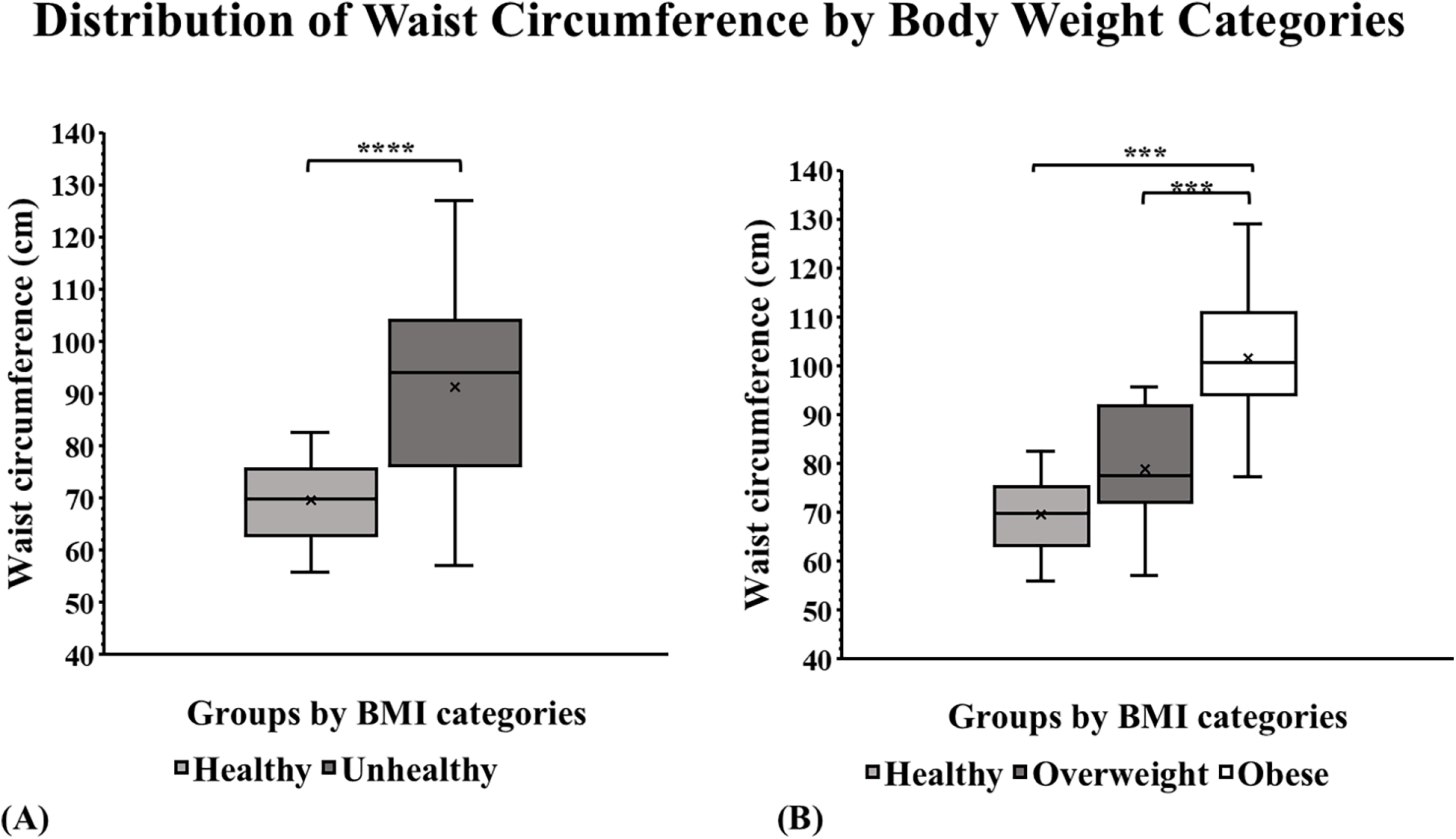
Anthropometry by BMI group categories in a sample of children from Puerto Rico. **(A)** The box plots showed the largest waist circumference among children with unhealthy weight. (*p* ≤ 0.001). **(B)** WC of the children with obesity was significantly larger compared to the healthy-weight group (*p* = 0.001) and overweight group (*p* = 0.001). *p ≤ 0.05, **p ≤ 0.01, ***p ≤ 0.001. ****p ≤ 0.0001.

The mean waist circumference (WC) in the healthy and unhealthy groups was 69.7 cm and 91.3 cm, respectively. The unhealthy group had a larger waist circumference when compared to the healthy group (*p* < 0.001) (Figure 1A**)**. Stratification of the unhealthy group into overweight and obese showed that the mean waist circumference was 78.9 cm and 101.5 cm, respectively. The obese group had a significantly larger waist circumference compared to both the healthy group (*p* = 0.001) and the overweight group (*p* = 0.001) (Figure 1B).

### 3.4 Liver ultrasound

#### 3.4.1 Liver size

**Figure 2:**
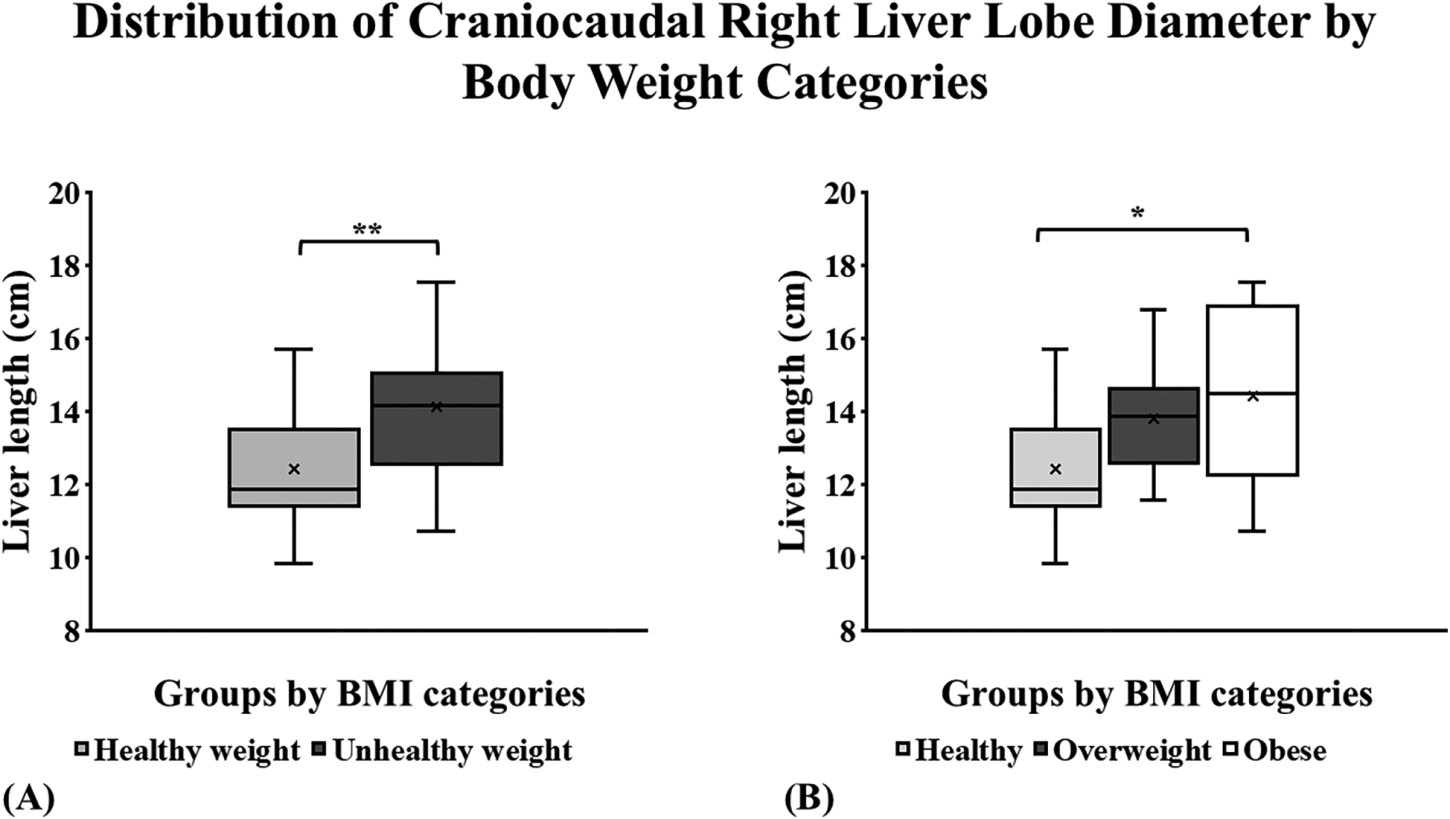
Craniocaudal panoramic right liver lobe (RLL) measurement distribution by BMI group categories in a sample of children from Puerto Rico. **(A)** The largest right liver lobe diameter was noted among children with unhealthy weight (*p* = 0.003). **(B)** The RLL of the children with obesity was significantly larger compared to the healthy-weight group (*p* = 0.02). *p ≤ 0.05, **p ≤ 0.01, ***p ≤ 0.001. ****p ≤ 0.0001.

The craniocaudal panoramic RLL measurements were used to evaluate and compare each group of subjects' liver sizes (Supplementary Table 1). The mean liver size for healthy weight was 12.4 cm, and for unhealthy weight was 14.1 cm. A significant difference was found in liver size across BMI categories between healthy and unhealthy weight groups (*p* = 0.003; Figure 2A). The stratification of the unhealthy weight group showed an average RLL diameter of 13.8 cm for the overweight and 14.4 cm for the obese. A Tukey test of these previously mentioned groups showed a significant difference between the RLL of healthy weight and obese groups (*p* = 0.02; Figure 2B). Pearson's correlation analysis between RLL and BMI revealed a weak-to-moderate positive correlation in the healthy weight group (*r* = 0.4281, *p* = 0.046), suggesting a statistically significant association. However, no significant correlation was observed in the overweight (*r* = 0.21, *p* = 0.56) and obese (*r* = 0.21, *p* = 0.54) groups, indicating that liver size variation was not strongly linked to BMI within higher BMI categories.

**Figure 3:**
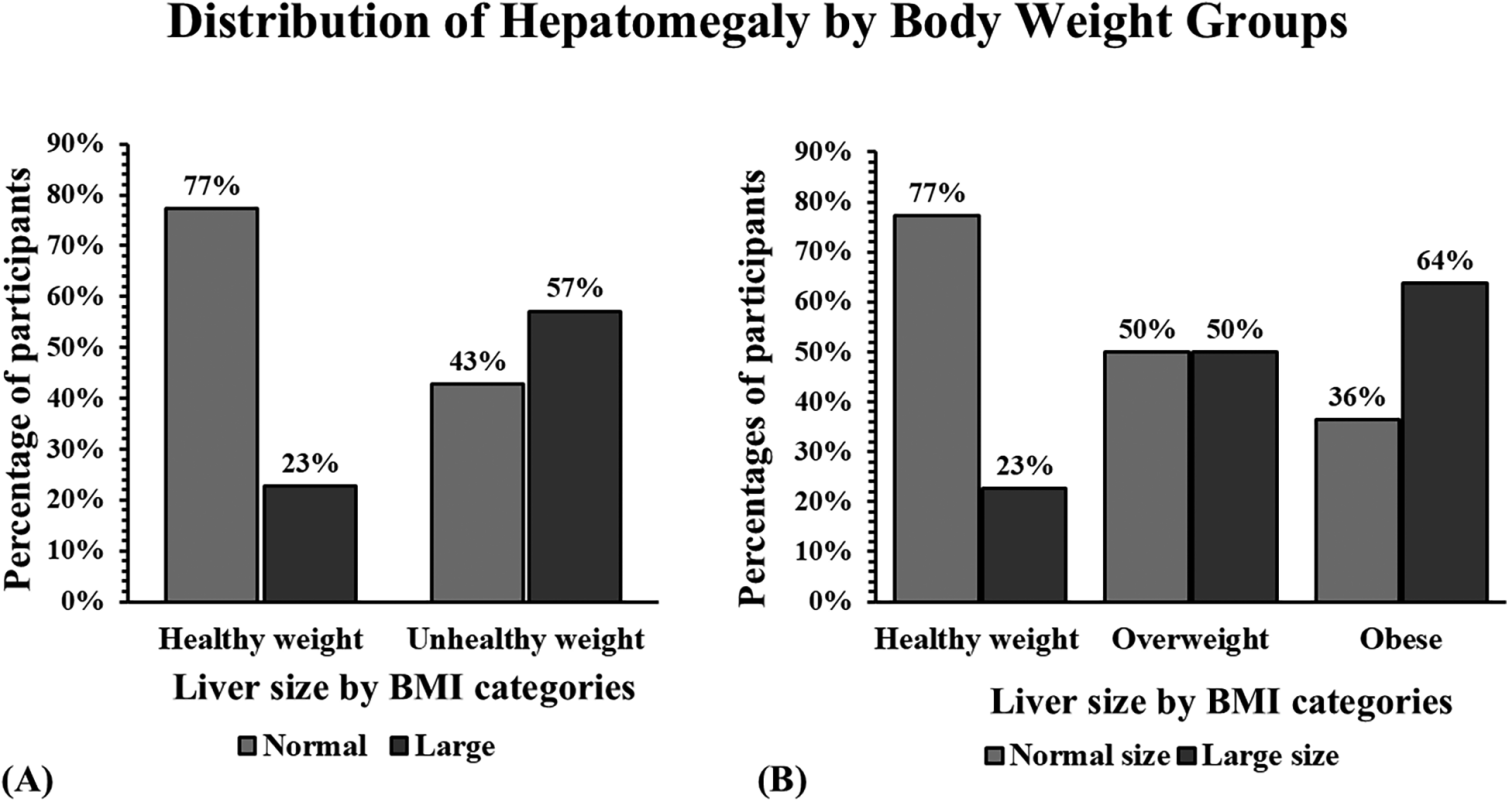
Distribution of hepatomegaly by BMI group categories in a sample of children from Puerto Rico. **(A)** Bar graphs show that the largest number of participants with hepatomegaly were children with unhealthy weight. **(B)** Children with obesity showed more cases of hepatomegaly than the other groups.

The distribution of hepatomegaly (18) (large liver size) among study participants was assessed following their body weight groups (Figure 3A). Dividing the unhealthy group into children with overweight and children with obesity provided more data about this participant sample. The overweight group had an exact 50/50 distribution concerning normal and large liver size (Figure 3B). In contrast, 36% of the children with obesity showed a normal liver size, and 64% of them had hepatomegaly (Figure 3B).

#### 3.4.2 Liver texture

**Figure 4:**
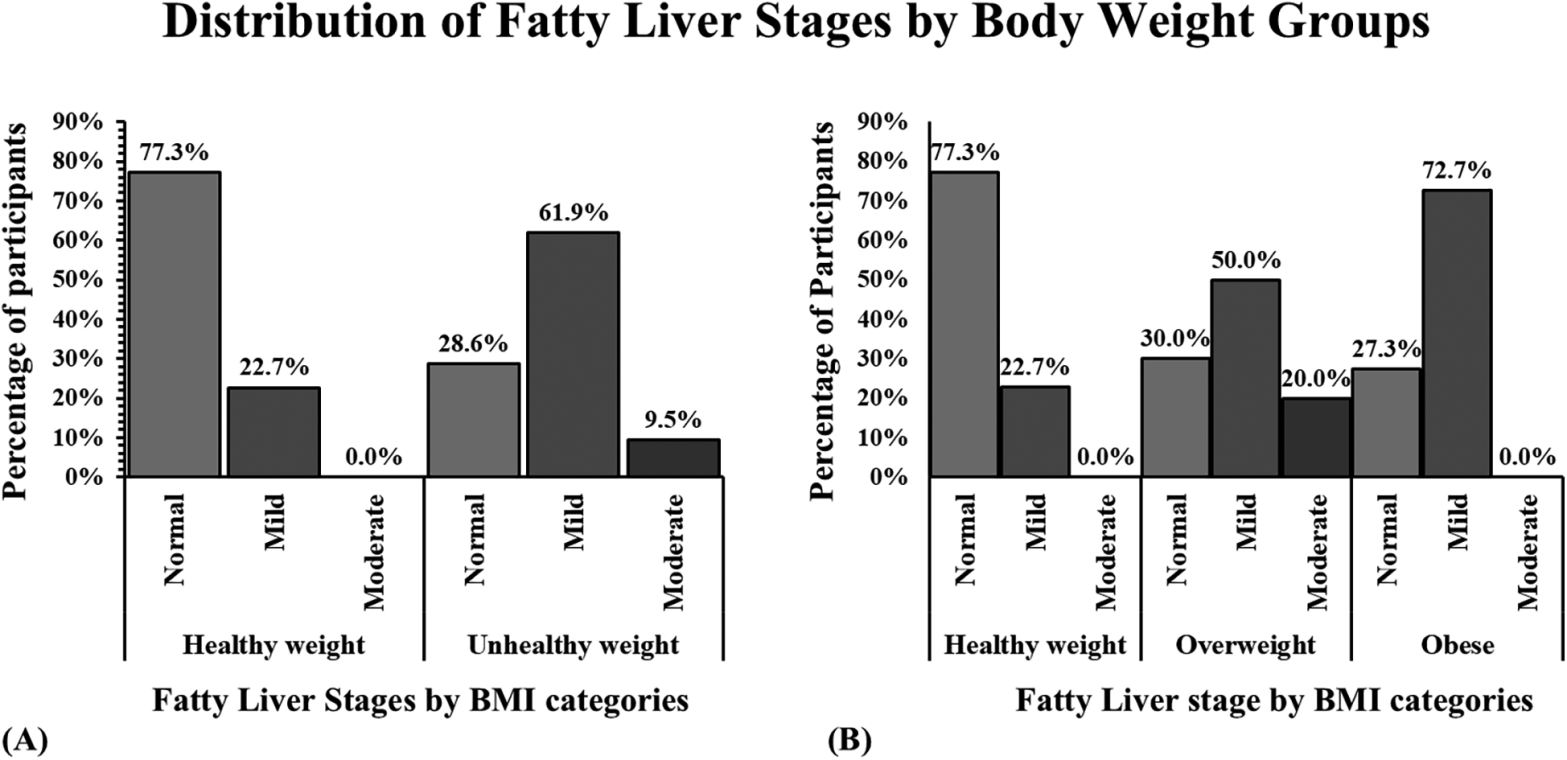
Hepatic fatty infiltration distribution by group categories in a sample of children from Puerto Rico. **(A)** The bar graph shows that children with unhealthy weight (overweight and obese) presented more livers with mild and moderate fat infiltration. **(B)** Children with obesity present a higher percentage of the liver with fatty infiltration.

Rumack test was performed to assess liver texture. Each participant was assigned a fatty stage that ranged from normal to moderate according to the Rumack scale t. In the healthy group, 77.3% had a normal fatty liver stage, while the other 22.7% had a mild ranking in this category. In the unhealthy group, 28.6% presented a normal fatty liver stage, 61.9% presented a mild stage, and 9.5% had a moderate stage (Figure 4A). The distribution of fatty liver grade was explored further in the unhealthy group by dividing them into overweight or obese subjects. Among the overweight subjects, 30% presented normally, 50% presented mildly, and the other 20% presented with a moderately fatty liver. Lastly, 27.3% of the obese participants presented a normal liver; the rest showed mildly fatty liver (72.7%; Figure 4B). The Chi-Square Test for Independence evaluated the association between BMI categories and fatty liver stage. The results indicated a statistically significant relationship (*p* = 0.0031), suggesting that liver texture distribution differs across BMI groups. Specifically, participants in the unhealthy weight group exhibited a higher prevalence of fatty liver changes compared to those in the healthy weight group.

## 4 Discussion

Childhood overweight and obesity prevalence has been increasing globally over the past three decades, becoming a public health problem (2). In our study of children from Puerto Rico, we found that large liver size and MAFLD were common among those with a BMI that falls at or above the 85th percentile for children and teens of the same age and gender. The findings of this study underscore the significant relationship between body weight and liver health in children from Puerto Rico, as evaluated through sonographic measurements. The analysis revealed a clear distinction in liver size and texture between children with healthy weight and those with unhealthy weight (overweight and obese), highlighting the potential of ultrasound as a non-invasive diagnostic tool for the early detection of liver abnormalities associated with MAFLD.

Children with an unhealthy weight had a waist circumference 1.36 times larger than healthy. This measurement is closely linked to abdominal obesity and fatty liver disease (19). Previous research indicates that an increase in children's waist circumference heightens the risk of developing conditions such as diabetes, cardiovascular disease, and liver fibrosis (20, 21). Our findings emphasize the significance of waist sizes in highlighting the increased risk for chronic diseases that these children may face in the future. This additional evidence further bolsters our findings regarding the potential impact of overweight and obesity on liver health in the children population.

To determine the liver size, our study implemented the measure established by Riestra et al., (2018) for the first time in the Puerto Rican children population (22). The impact of increased BMI was evident when measuring the participant's craniocaudal panoramic right liver lobe. Healthy-weight participants had an average liver lobe diameter of approximately 12.5 cm compared to 14.25 cm in unhealthy-weight participants. Participants with an unhealthy weight had a liver lobe diameter 1.1× bigger than those considered healthy. A systematic review in 2019 found that the average craniocaudal liver size in children measured using ultrasound ranged from 7 cm in young children to a maximum of 12.1 cm in older children (23). Our study findings show liver sizes that surpass these measurements, emphasizing how obesity could be contributing to larger liver sizes.

Similar findings were obtained when distributing normal vs. large liver sizes by body weight groups (19). The most significant number of children with hepatomegaly or enlarged liver were those among the unhealthy-weight sample. In fact, 57% of those classified as unhealthy weight had a large liver size compared to only 23% of those classified as healthy, for an increase of 2.5× in liver size. In previous studies, hepatomegaly has been found in children with healthy weight due to lysosomal acid lipase deficiency, inappropriate glycogen storage, metals, toxins, infections, vascular congestion, and biliary obstruction (24–26). This finding was also significant when stratifying according to BMI categories, with those considered obese having a liver size 2.8× larger than those considered having a healthy weight. Given the potential link between hepatomegaly and liver distress, it is essential to conduct further research to understand how liver size in childhood correlates with the prevalence of chronic diseases in adulthood. However, studies do show that liver size can have a clinical impact in managing diseases like hepatosteatosis, portal hypertension, liver disorders, and cardiac diseases that lead to hepatopathy (27, 28). Thus, it is essential to identify and address those who are unhealthy, overweight, and obese in the children population to decrease the risks that hepatomegaly can cause in adulthood and limit the incidence of chronic diseases that may arise as a consequence of hepatomegaly.

The results of this research not only suggested a connection between increased waist size and liver size but also revealed a greater occurrence of fatty infiltration in children with obesity. Fatty liver onset and advancement during childhood are prone to elevate the likelihood of enduring health complications in the future, like liver fibrosis, diabetes mellitus, cardiovascular disease, and cancer (29–31). Therefore, our results indicated that fatty liver changes are more frequent in children with obesity and are occurring among children, echoing other similar study findings (3, 19). In our sample, 61.9% of children with unhealthy weight had mild fat infiltration while 9.5% had moderate fat infiltration; therefore, two-thirds of our participants classified as unhealthy weight had evidence of fat infiltration. When analyzing the percentages categorized by BMI, children classified as obese had 72.7% mild fat infiltration, further highlighting that the majority of obese children had fat infiltration. Interestingly, individuals classified as overweight exhibited the highest percentage of moderate fat changes (20%), while those categorized as obese showed no such changes. These findings support the hypothesis that increased BMI is associated with early liver texture alterations detectable via ultrasound. Therefore, additional investigation is required to elucidate the factors driving moderate fat alterations, irrespective of weight.

Nevertheless, these results emphasize that fatty infiltration, the hallmark of MAFLD, is occurring among children at an alarming rate. While MAFLD is asymptomatic until the late stages (16), fat infiltration in the liver has been associated with alterations in glucose, fatty acid, and lipoprotein metabolism, potentially leading to the development of insulin resistance, dyslipidemia, and cardiovascular disease (32). A study found that children with MAFLD were at increased risk of progressing to nonalcoholic steatohepatitis or NASH, increasing their risk for significant morbidity caused by cirrhosis (33). Therefore, children from Puerto Rico suffering from obesity could be at an increased risk for liver and metabolic diseases compared to children with healthy weight.

While limited data exists on the long-term effects of children's MAFLD in adulthood, our findings highlight the importance of implementing preventative and screening methods to help mitigate the consequences of MAFLD. In children, MAFLD is usually found incidentally via abdominal imaging, as there are no routine screening methods. Integrating screening methods for children with unhealthy weight might allow for early intervention and contribute to decreasing the risk of liver disease consequences in adulthood. Additionally, studies have found that prevention via family and community education regarding nutrition can be successful in reducing the incidence of MAFLD in children (16, 34). Also, lifestyle interventions via exercise integration of 60 min three times a week have been shown to reduce liver fat content and improve cardiovascular health in children (16). Approaches in exercise implementation and community education could help mitigate the incidence of MAFLD in children. Thus alleviating the potential burden associated with chronic diseases in the healthcare system.

Although this study illustrates the incidence of hepatomegaly and liver fatty infiltration in a sample of children in Puerto Rico, there are some limitations to consider. Even though the database accounts for current waist circumference, height, and weight, there is no way of knowing if any patient with a normal or underweight BMI was previously overweight or vice versa and how fluctuating weight changes affect liver health. Additionally, our study sample size was 43 children between the ages of 7 and 19. The comparison of variables such as liver enzymes, blood pressure, insulin resistance, and lipoprotein metabolism could also be reconsidered. While our findings were significant, future studies with larger sample sizes could be developed to confirm them.

Data from the CDC shows that 19.7% of the pediatric population are children and adolescents with obesity (35). Nationally, MAFLD is considered to be the most common cause of liver disease in children, and its incidence is expected to rise with the constant increase in obesity (33). A previous systematic review showed that dietary nutrients play an important role in the onset and progression of MAFLD (36). Even though biopsy remains the gold standard method for evaluating liver tissue, it is highly invasive with high risks of complications and adverse events and high costs. Liver biopsy has the benefit of being able to detect >5% fat accumulation in hepatocytes (37). On the other hand, ultrasound is a noninvasive tool used in recent years to perform liver imaging and detect changes in liver tissue caused by fat infiltration. Although it has limitations in that it usually detects >20%–30% fat accumulation in hepatocytes, multiple ultrasound tools have been developed (Ultrasound Attenuation Imaging (ATI—for Quantitative assessment of hepatic fat); Controlled Attenuation Parameter (CAP, FibroScan®—to measure liver fat accumulation, and Quantitative Ultrasound (QUS- uses backscatter and attenuation to estimate steatosis) that allow for effective and noninvasive evaluation of quantitative fat accumulation in hepatocytes (38). This makes it an excellent tool, especially among children, where noninvasive procedures are preferred. Nevertheless, the results from this study highlight the significant consequences that obesity can have on children's liver health and the importance of understanding the needs and impact on the future well-being of the pediatric population. Additionally, longitudinal studies are needed to examine the health effects of hepatomegaly and fatty infiltration in children as they transition into adulthood. Additionally, it's important to investigate screening methods that can be incorporated into pediatric health practices for early evaluation and intervention.

There is extensive evidence highlighting the physical, psychological, and economic toll of obesity-related complications, along with an increased risk of cardiometabolic diseases, morbidity, and mortality. Many of these complications are preventable with early evaluation and intervention. It is crucial to implement strategies aimed at preventing unhealthy weight in children to mitigate the burden associated with chronic diseases and potential disabilities in young adults. Early intervention is key to averting these health risks.

## Data Availability

The raw data supporting the conclusions of this article will be made available by the authors, without undue reservation.
