# Supplementary material for: Ultrasound assessment of hepatomegaly and metabolically-associated fatty liver disease among a sample of children: a pilot project
Source: Front Pediatr. 2025 Apr 28;13:1491342. doi: 10.3389/fped.2025.1491342 (PMC12066509; doi:10.3389/fped.2025.1491342)
Supplement: Supplementary file 1 [file Table1.docx]

Supplementary Material

# Supplementary Tables

**Supplementary Table 1. Distribution of Right Liver Lobe (RLL) measurement by age range.** The table provides each patient's RLL measurements by age range according to BMI categories and the ranges of normal RLL values ​​for each age range.

| **7-9 years**  **Normal length range: 7.5 – 13.0 cm** | | **10-11 years**  **Normal length range: 7.5 – 13.5 cm** | |
| --- | --- | --- | --- |
| **Healthy RLL (cm)** | **Unhealthy RLL (cm)** | **Healthy RLL (cm)** | **Unhealthy RLL (cm)** |
| 11.4 | **17.3** | **14.0** | 11.8 |
| 10.7 | **12.6** | 11.7 | **15.4** |
| 11.5 | **14.6** | 12.0 | **14.1** |
| 9.8 | 12.3 |  |  |
| 10.7 |  |  |  |
| **12-15 years**  **Normal length range: 8.5 – 14.0 cm** | | **16-19 years**  **Normal length range: 9.5 – 14.5 cm** | |
| **Healthy RLL (cm)** | **Unhealthy RLL (cm)** | **Healthy RLL (cm)** | **Unhealthy RLL (cm)** |
| 12.8 | 17.5 | 10.7 | 13.3 |
| 13.2 | 13.1 | 11.5 | 14.5 |
| 11.7 | **14.2** | 11.8 | 12.5 |
| **15.7** | **16.9** | **14.8** | **14.6** |
| 13.3 | **14.7** | 12.7 | **16.8** |
| 11.4 | 13.6 | **14.9** | 10.7 |
|  | 11.6 | **14.8** |  |
|  | **14.5** | 12.7 |  |

**Bold** = RLL larger

Reference:

The Radiology Assistant: Normal Values in Ultrasound [Internet]. radiologyassistant.nl. Available from: https://radiologyassistant.nl/pediatrics/normal-values/normal-values-ultrasound
